# Long-term outcome and effect of maintenance therapy in patients with advanced sarcoma treated with trabectedin: an analysis of 181 patients of the French ATU compassionate use program

**DOI:** 10.1186/1471-2407-13-64

**Published:** 2013-02-06

**Authors:** Jean-Yves Blay, Antoine Italiano, Isabelle Ray-Coquard, Axel Le Cesne, Florence Duffaud, Maria Rios, Olivier Collard, François Bertucci, Emmanuelle Bompas, Nicolas Isambert, Loic Chaigneau, Philippe Cassier, Binh Bui, Gauthier Decanter, Olfa Derbel, Jean-Michel Coindre, Patrick Zintl, Nadia Badri, Nicolas Penel

**Affiliations:** 1 Centre Leon Berard, Lyon, France; 2 Institut Bergonie, Bordeaux, France; 3 Institut Gustave Roussy, Villejuif, France; 4 Hopital de La Timone, Marseille, France; 5 Centre Alexis Vautrin, Nancy, France; 6 Institut de Cancérologie de la Loire, Saint Etienne, France; 7 Institut Paoli-Calmettes Calmette, Marseille, France; 8 Centre René Gauducheau, Nantes, France; 9 Centre GF Leclerc, Dijon, France; 10 CHU Besançon, Besançon, France; 11 Pharmamar, Madrid, Spain; 12 Centre Oscar Lambret, Lille, France

## Abstract

**Background:**

The long term outcome of advanced sarcoma patients treated with trabectedin outside of clinical trials and the utility of maintenance treatment has not been reported.

**Methods:**

Between 2003 and 2008, patients with advanced sarcoma failing doxorubicin could be treated within a compassionate use program (ATU, Temporary Use Authorization) of trabectedin in France using the standard 3-weekly regimen. Data from 181 patients (55%) were collected from 11 centres and analyzed.

**Results:**

Trabectedin was given in first, second, third or fourth line in metastatic phase in 6%, 37%, 33% and 23% of patients respectively. With a median follow-up of 6 years, median PFS and OS were 3.6 months and 16.1 months respectively. The median number of cycles was 3 (range 1–19). Best response were partial response (PR, n = 18, 10%), stable disease (SD, n = 69, 39%) and progressive disease (PD, n = 83, 46%), non evaluable (NE, n = 9, 5%). Thirty patients (17%) had to be hospitalized for treatment- related side effects. Independent prognostic factors in multivariate analysis (Cox model) were myxoid LPS and line of trabectedin for PFS, and myxoid LPS and retroperitoneal sarcomas for OS. Patients in PR or SD after 6 cycles continuing treatment had a better PFS (median 5.3 vs 10.5 months, p = 0.001) and OS (median 13.9 vs 33.4 months, p = 0.009) as compared to patients who stopped after 6 cycles.

**Conclusions:**

In this compassionate use program, trabectedin yielded similar or better PFS and OS than in clinical trials. Maintenance treatment beyond 6 cycles was associated with an improved survival.

## Background

Soft tissue sarcoma (STS) constitutes a heterogeneous group of rare cancers, with heterogeneous clinical presentation, histological subtypes and molecular alterations [1]. The established standard of care for unresectable STS in first line is doxorubicin-based chemotherapy, with typical response rates ranging from 10% to 30% [1-4]. For patients who relapse or develop resistance, other therapeutic options were limited before the availability of trabectedin [5]. For these patients, progression-free survival (PFS) and overall survival (OS) rarely exceed 6 months and 1 year respectively [5].

Trabectedin is a tetrahydroisoquinolone alkaloid isolated from the marine organism *Ecteinascidia turbinata*, a tunicate originally extracted from the Caribbean Sea. Its complex mechanism of action involves a covalent bond to the minor groove of double-stranded DNA, resulting in an inhibition of gene activation and nucleotide excision repair (NER) mechanism, and also inducing lethal DNA double-strand breaks and cell cycle arrest in S and G2 phases [6-11]. *In vitro*, trabectedin has shown potent cytotoxic activity against a variety of human STS cell lines, and antitumor activity against a variety of human xenografts, including sarcomas, with limited cross-resistance between trabectedin and other cytotoxic agents [11-15].

In clinical trials, single-agent trabectedin has shown activity in a variety of tumor types, including sarcomas, breast cancer, and ovarian cancer [16-28]. The clinical activity of single-agent trabectedin has been demonstrated in heavily pretreated patients with advanced STS, with a median duration of response of 9 to 12 months and 6-months PFS rates ranging from 24% to 29% [16-24], as well as in first line patients, either as single agent or in combination with doxorubicin [25,26]. The STS201 randomized, open-label study was conducted in adult STS patients with unresectable/metastatic liposarcoma or leiomyosarcoma, after failure of prior conventional chemotherapy including anthracyclins and ifosfamidei. Patients were randomly assigned to one of two trabectedin regimens (given intravenously at a dose of 1.5 mg/m^2^ on a 24-h infusion every 3 weeks or at a dose of 0.58 mg/m^2^ on a 3-h infusion weekly for 3 weeks of a 4-week cycle). The study met its primary endpoint with a median TTP of 3.7 months in the 24-h arm vs. 2.3 months in the 3-h arm (p = 0.0302), showing a statistically significant 27% reduction in the risk of progression with the 24-h trabectedin arm [27]. According to these results, trabectedin was approved in September 2007 in the European Union for patients with advanced STS after failure of anthracyclins or ifosfamide or for those who are unsuited to receive such agents. Before that date, a compassionate use program (ATU) was set up in France where 328 patients were included since 2003. The efficacy of trabectedin in compassionate use programs may be different from those obtained in clinical trials, because patients with less favorable clinical characteristics are included.

Hereby are reported the results of a retrospective study in which the outcome of patients included in this compassionate use program population was analyzed. The survival and response rates of these patients were comparable to those reported in clinical trials. Interestingly, maintenance treatment after 6 cycles was associated with improved PFS and OS over treatment discontinuation after 6 cycles.

## Methods

### Centres

From 2003 to 2008, 87 centres in France enrolled included at least one patient in the ATU (“Autorisation Temporaire d’Utilisation”) program, a compassionate use program for STS patients matching the inclusion criteria (see below). Requests for participation were sent to all 43 centres that included more than 1 patient. Only centres that had included more than 5 patients actually contributed to this retrospective study. These centres treated 252 patients in total, among which 181 patient files (71%) were collected and updated as of March 20^th^, 2012. 181 of the 328 (55%) patients of the ATU program are therefore included in this report. Trabectedin was given at the standard schedule of 1.5 mg/m2 in 24 h continuous infusion every 21 days, as previously reported, with dose adaptations similar to those applied in the protocols [19-23].

#### Objectives

The primary objective of this study was to evaluate progression-free survival, while secondary endpoints were response rates, duration of response, overall survival, toxicity leading to hospital rehospitalisation, description of the patient populations, impact of treatment duration on treatment efficacy. Because of its retrospective nature, only very simple clinical parameters were collected.

### Inclusion criteria for the retrospective study

These criteria were those from the EORTC trial [23], the largest of the single-arm phase II studies with trabectedin. Patients had to have a documented progressive disease at inclusion). No concurrent antitumor therapy was allowed. Other eligibility criteria were age older than 18 years; performance status 0 or 1; no functionally important cardiovascular disease, no prior cancer (except adequately treated *in situ* carcinoma of cervix or basal cell carcinoma); presence of measurable lesions not previously irradiated, no central nervous system metastases; adequate bone marrow reserve (neutrophils > 2,000/mm^3^, platelet count > 100,000/mm^3^); and adequate renal and hepatic functions: serum creatinin less than 120 μmol/L or calculated creatinin clearance (Cockroft method) greater than 60 mL/min, bilirubin > 30 μmol/L, AST and ALT less than 1.5 U/L (<2.5 U/L in case of liver metastases), alkaline phosphatase less than 2.5 U/L and albumine > 25 g/L. Mesothelioma, chondrosarcoma, neuroblastoma, osteosarcoma, Ewing’s sarcoma, embryonal rhabdomyosarcoma, and dermatofibrosarcoma were excluded.

### Case report form

A simple Case Report Form with 22 items was used to collect patients’ characteristics and outcome. Information has been collected on an excel spreadsheet, consolidated in an Excel database, and then analyzed using the SPSS 12.1 software by institutional data manager. Collected information included the following: anonymized patient identity, centre, date of birth, gender, date of diagnosis, histotype, grade, date of metastasis, description of first/s/>2 line treatments, best response, duration, date of trabectedin first course, ECOG PS at that date, metastatic sites at that date (lung, liver, local, soft part, bone, or other), number of cycles, best response to trabectedin, toxicity of trabectedin requiring re-admission, date of last course of trabectedin, date of progression after trabectedin, treatment after trabectedin and best response, date of death. Optional data were number of available pathology tissue block, and contact information for the pathology department where diagnosis was made and which held the pathology samples.

### Descriptive analysis and statistics

Baseline demographics and clinical outcome statistical analyses were based on all data available up to the cut-off date of December, 31^st^ 2011. Descriptive statistics were used to depict the variables distribution. Follow-up was calculated from course 1 of trabectedin. Progression-free survival (PFS) was defined as the interval between the date of the first trabectedin cycle and the date of disease progression, death, or last follow-up contact. The interval between the date of the first cycle of trabectedin and the time of death or last follow-up defined the Overall Survival (OS). PFS and OS rates were estimated using the Kaplan-Meier method and were compared using the log-rank test. Univariate analyses included the following variables: age; sex; performance status; grade; histological subtype; disease location, myxoid liposarcoma histology, translocation sarcoma, treatment line, hospitalization for toxicity and liver/lung metastases. Responses were determined retrospectively using RECIST 1.1. All statistical tests were 2-sided, and a p-value below 0.050 was considered statistically significant. This study was approved by the local institutional review board at each participating institution.

## Results

### Population and patient characteristics

Between 2003 and 2008, 87 centres have included 328 patients in the ATU program. The present study was performed on 181 patients from 11 centres having treated at least 5 patients who agreed to participate. This represents 55% of the total cohort of 328 ATU patients. Inclusion criteria of the ATU were those of the EORTC trial. The only difference was that no restrictions were imposed on the previous number of lines. The median number of patient per centre was 17 (range 5 to 32). Patients’ characteristics are described in Table 1. 29% had translocation-related sarcomas. At diagnosis, grade 3, 2 and 1 STS represented 44%, 28% and 9% of the tumors respectively. Median line of therapy was third line for trabectedin, with a range of 1 to 4 lines. 56 of 181 patients (31%) received 6 cycles or more. During the course of treatment, 30 (17%) of the patients had to be re-admitted for treatment-related adverse events.

**Table 1:** Patients characteristics and survival

		**N (%)**	**Median OS**		**Median PFS**	
			**months**	**logrank**	**months**	**logrank**
**Age**		47 (15–84)				
	<=60	145 (80)	16.9	0.05	3.3	0,13
	>60	36 (20)	11.7		3.0	
**Male**		83 (46)	14,7	0.36	3.8	0,78
**Female**		98 (54)	16,6		3.1	
**Site**						
	Inf limb	59 (33)	17.1	0.15	4.8	**0.03**
	Sup Limb	12 (7)	16.6		**2.3**	
	Retroperitoneum	33 (18)	19.6		**9.1**	
	Uterus	24 (13)	13.9		**3.0**	
	Other trunk	45 (25)	9.3		**2.7**	
	Head/Neck	8 (4)	17.0		**4.0**	
**Histotypes**						
	LMS	55 (30)	17,4	**0.005**	3.4	**0.005**
	Myxoid LPS	28 (16)	33.4		10,5	
	LPS (other)	21 (11)	20.0		**3.2**	
	NOS/undiff.	22 (12)	14,0		2.2	
	Synovial	16 (9)	9.2		**4.0**	
	Others/Misc	39 (22)	6.7		**2.1**	
**Myxoid LPS**						
	Yes	28 (15)	33.4	**0.01**	10,5	**0.000**
	No	153 (85)	13.9		2.8	
**Translocation sarcomas**						
	Yes	54 (30)	15.3	0.17	5.3	0,02
	No	119 (66)	14.7		2.8	
**Grade**						
	1	17 (9)	16.9	0.43	9.7	0,17
	2	51 (28)	18.6		4.4	
	3	80 (44)	16.0		3.6	
	UNK	33 (19)	10.7		2.8	
**Treatment line for ET-743***(median: 3, range 1–4)*						
	1*	10 (6)	33.4	**0.03**	4.5	**0.04**
	2	67 (37)	18.2		5.3	
	3	60 (33)	14.3		3.2	
	4	42 (23)	10.2		2.8	
* doxorubicin and ifosfamide in the adjuvant setting						
**Hospitalisation for toxicity***(reported in N = 152)*						
	Yes	30 (17)	6.7	**0.03**	2.5	0.39
	No	122 (65)	18.2		4.5	
**Lung metastases**						
	Yes	128 (71)	14.7	0.93	3.0	0.054
	No	47 (25)	16.9		6.7	
**Liver metastases**						
	Yes	45 (25)	16.8	0.51	3.0	0.83
	No	125 (70)	13.5		3.6	

### Survival

**Figure 1:**
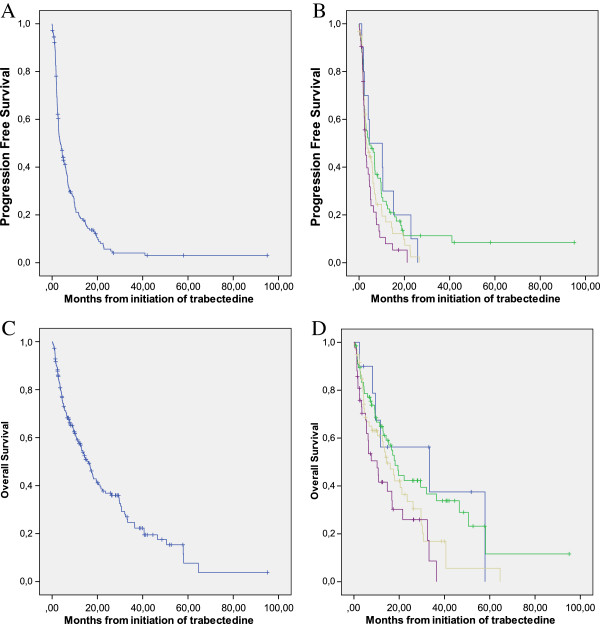
**Progression-free and overall survival of the cohort according to the number of lines. A**: Progression-free survival in the whole cohort. **B**: Progression-free survival of patients treated in first (blue), second (green), third (light brown) or >3 (purple) line of treatment (log-rank, p = 0,043). **C**: Overall survival in the whole cohort. **D**: Overall survival of patients treated in first (blue), second (green), third (light brown) or >3 (purple) line of treatment (logrank, p = 0,018).

With a median follow-up of 64 months after initiation of trabectedin treatment, the median PFS was 3.6 months, with a 6-months PFS rate of 39%. Median OS survival was 16.1 months, with 3, 4, and 5 years OS rate of 23%, 15%, and 4% respectively (Figure 1A and 1C). PFS and OS were superior in patients treated in 1^st^ and 2^nd^ line but prolonged survival >24 months was observed in all subgroups (Figure 1B, and 1D). PFS was superior in patients with myxoid liposarcomas, retroperitoneal sarcomas and grade 1 tumors (Table 1). OS was superior in patients with myxoid liposarcomas, retroperitoneal sarcomas and grade 1 tumors (Table 1). In multivariate analysis, the only two independent prognostic factors identified for PFS were histological subtype of myxoid LPS and the line of treatment. For OS, the two favorable prognostic factors in multivariate analysis were histological subtype of myxoid liposarcomas and retroperitoneal locations for primary disease (Table 2).

**Table 2:** Multivariate prognostic analysis for PFS and OS

		**beta**	**E.S.**	**Signif.**	**HR**
PFS	Myxoid LPS	−0.544	0.284	0.056	0.580
	Line of treatment	0.272	0.117	0.020	1.312
OS	Myxoid LPS	−0.970	0.241	0.000	0.379
	Retroperitoneal STS	−0.524	0.209	0.012	0.592

### Response to treatment

**Figure 2:**
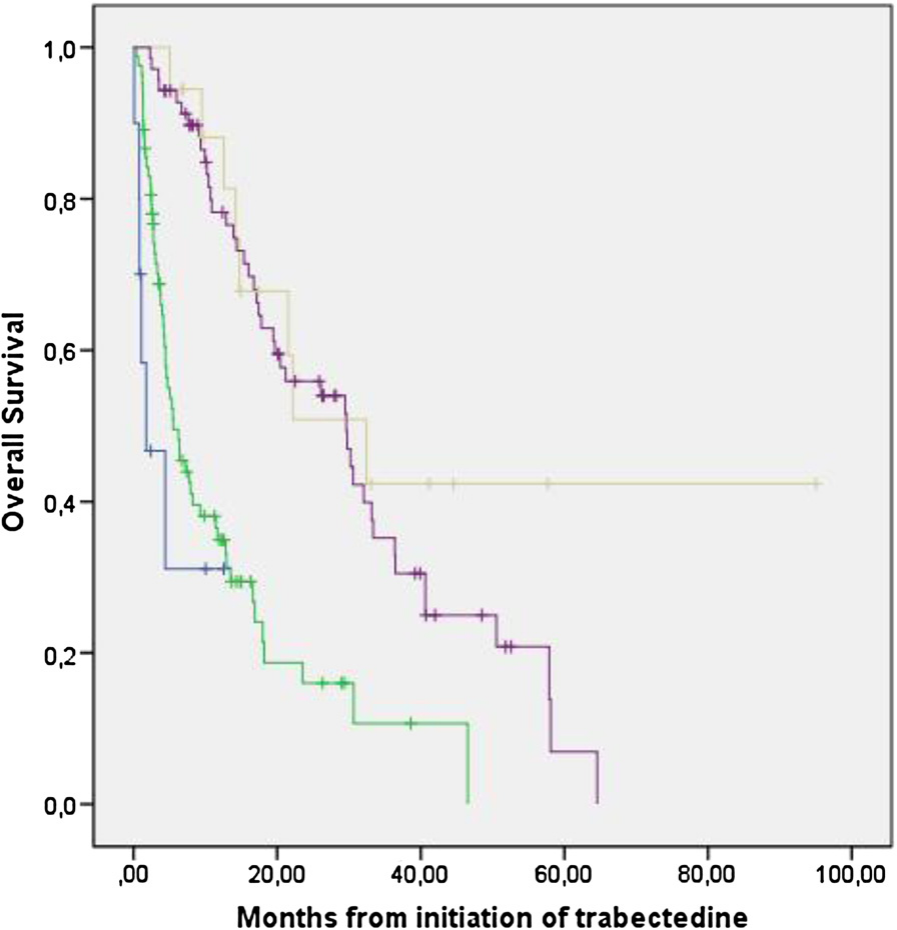
**Overall survival according to the best response to trabectedin.** Overall survival of patients whose best response was: partial response (light brown), stable disease (purple), progressive disease (green), or non evaluable (blue). Log-rank p value, p < 0,0001.

Partial response (PR), stable disease (SD), and progressive disease (PD) were recorded as best response in 10%, 39%, 46% of the patients respectively, with 5% patients being non evaluable. No significant difference was were observed according to the line of trabectedin administration (Table 3, p = 0.17). Myxoid liposarcoma had a better response and stable disease rate (21% and 54% respectively) as compared to other histological types (8% and 36% respectively) (p = 0,002) with no significant difference between other translocation-related sarcomas and the remaining group of sarcoma (not shown). The median duration of response was 10.5 months (95% CI: 5.4–15.6). Overall survival of partial responders and patients with SD were similar in the first years, but only partial responders were long-term survivors beyond 5 years (44%). Overall survival of patients with PR or SD subgroups, were equivalent, and both superior to that of patients with progressive disease or non evaluable disease as best response (Figure 2).

**Table 3:** Response to trabectedin according to the line of treatment

			**Best response to trabectedine**				**Total**
			**NE**	**PD**	**SD**	**PR**	
**Line of treatment**	**1**	**N**	**0**	**3**	**4**	**3**	**10**
		**%**	**,0%**	**30,0%**	**34,0%**	**30,0%**	**100,0%**
	**2**	**N**	**1**	**32**	**26**	**8**	**67**
		**%**	**1,5%**	**47,8%**	**38,8%**	**11,9%**	**100,0%**
	**3**	**N**	**4**	**26**	**27**	**3**	**60**
		**%**	**6,7%**	**43,3%**	**45,0%**	**5,0%**	**100,0%**
	**4**	**N**	**4**	**22**	**12**	**4**	**42**
		**%**	**9,5%**	**52,4%**	**28,6%**	**9.5%**	**100,0%**
**Total**		**N**	**9**	**83**	**69**	**18**	**179**
		**%**	**5,0%**	**46,4%**	**38,5%**	**10,1%**	**100,0%**

### Maintenance therapy after 6 cycles

**Figure 3:**
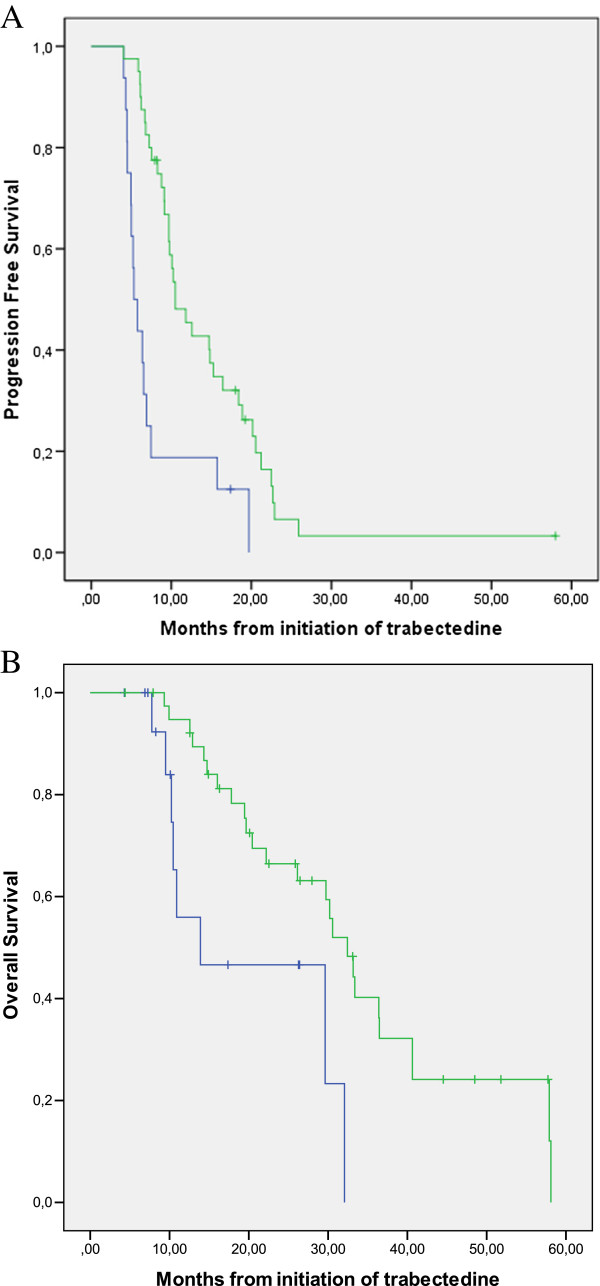
**Progression-free and overall survival according to maintenance after 6 cycles.** No maintenance- treatment interruption after 6 cycles (blue); maintenance- treatment beyond 6 cycles (green). Log-rank p-value for PFS, p = 0,007; Log-rank for OS, p = 0,0002.

A total of 56 (31.1%) patients were in SD or PR after 6 cycles. In 16, the treatment was stopped, whereas in 40 patients it was continued beyond 6 cycles for a median of 9 cycles (range 7–19). The subgroup of patients treated with 7 or more cycles had a significantly better PFS (median 5.3 months vs 10,5 months, p = 0,001) and OS (median 13,9 vs 33,4 months p = 0.009) (Figure 3) than the other subgroup, suggesting that maintenance). Maintenance therapy was associated with a better PFS and OS in this series analyzed retrospectively.

## Discussion

The objective of this retrospective study was to assess the outcome of STS patients treated in the French ATU compassionate use program and to compare it with that of published clinical trials. Between 2003 and 2008, this program enabled the treatment of patients failing doxorubicin with trabectedin 1.5 mg/m2/21d. The inclusion criteria were the same than those of the EORTC trial, with the exception that all lines were allowed.

Not all patients could be retrospectively collected. Actually only centres that included more than 5 patients contributed to this analysis, and these included 181 patients in this retrospective study. This series represents therefore a selected subgroup of patients treated mostly in reference centres for sarcoma, and in experienced centres regarding trabectedin usage. Among the 11 centres participating to the study, 5 had participated to the phase II EORTC trial, reflecting the experience of the centres with this agent. This is therefore a selected subgroup of the ATU series, but this selection makes comparison with phase II data maybe more relevant. It would have been of interest to compare this series to that of patients treated in non-expert centres but this could not be obtained. In this group of 181 heavily pretreated STS sarcoma patients, either resistant or relapsing, trabectedin was received as a second line therapy for a majority of them and some patients received the treatment in 4th line. This is a more heavily pretreated patient population than that of the EORTC trials. Despite of this, the response rate (10%), stable disease rate (39%), PFS (median 3.6 months) and OS (median 16.1 months) were comparable to those observed with trabectedin in the phase II trials. According to the EORTC-STBSG (European Organisation for Research and Treatment of Cancer- Soft Tissue and Bone Sarcoma Group) criteria, because the 3-months progression free rate was largely superior to 40% and the 6-monthz PFR was superior to the threshold used to define an active treatment according to the EORTC STBSG [5]. It is however challenging to compare the present series with the EORTC database of the pre-trabectedin era [2,3] published since 1999 for several reasons : 1) the former series included mainly first line patients, while the present series gathers patients in all lines (from first line metastatic in patients pretreated in the adjuvant setting to fourth line patients. 2) histological classifications and inclusion criteria varied considerably between the 2 series; for instance, GIST were mixed amongst leiomyosarcoma in the former series. The exhaustive histological reviews of the former series were has not performed with the classifications of 2002 or 2013.

Possibly the best comparison can be obtained with the subsequent paper by Van Glabbeke et al., reporting separately second line + patients. In this case, the median progression free rate is 2.3 months, and a 1 year PFR rate of 7% in the whole series, and 12% for the series of patients treated with “active agents” [5]. The results observed with the present ATU series, median PFS of 3,6 months, and 12 months PFS close to 30% compare therefore favorably with these historical controls, despite all these limitations.

Detailed side-effects of trabectedin treatment were not collected in this retrospective study. Only toxicity leading to hospitalization was documented and remained limited, affecting only 17% of patients. As expected these patients had a smaller number of cycles delivered and, perhaps as a consequence, had a worse PFS. Overall survival was however not significantly different than that of on patients without toxicity-related hospitalization. Because most toxicities do not lead to rehospitalisation, no formal conclusion can be proposed on a possible lack of correlation with therapeutic efficacy, considering also the limited number of patients in this series.

As previously described, patients with myxoid liposarcomas had better response rates, PFS,OS, and was an independent prognostic factor for survival. The number of lines of chemotherapy administered before trabectedin also correlated significantly with longer PFS in the Cox model, but not for OS. Conversely, retroperitoneal location was were associated with a improved survival, possibly because of the low grade and loco-regional behavior; most are liposarcomas, a subset associated with a better outcome in large retrospective datasets in the present series as well [2]. Interestingly, OS and PFS of translocation-related sarcomas excluding myxoid liposarcoma was not different of that of other sarcoma types. Similar observations were made for response rate (not shown).

Thirty percent of the patients in the present study received more than 6 cycles of trabectedin, which underlines an acceptable toxicity profile allowing prolonged treatment. Long-term treatment is feasible with trabectedin, while this is not feasible with doxorubicin nor ifosfamide because of cumulative cardiac and renal toxicities. Prolonged trabectedin treatment thus allows testing the importance of maintenance treatment. Interestingly, among the 56 patients who were not progressing after 6 cycles, the 40 who continued treatment had a significantly better PFS and more surprisingly OS, with a more than doubling of the median OS. The retrospective nature of the study implies potentials biases in these observations, and therefore this cannot be considered as an evidence for the utility of prolonged treatment. However, these observations strengthen the rationale of the ongoing study randomizing treatment maintenance vs interruption after 6 cycles which is currently ongoing within the French Sarcoma Group (NCT01303094). It has previously been shown in a large randomized clinical trial (SUCCEED) that maintenance with an mTOR inhibitor enables to prolong PFS, but not OS (Demetri et al. submitted for publication). Maintenance therapy maybe a strategy worth further exploring in patients with advanced STS.

## Conclusion

In conclusion, this retrospective analysis of 55% of the advanced sarcoma patients treated in France in the compassionate use program shows that the use of trabectedin in routine clinical practice, in large volume centres, yields an outcome similar to the previously observed results in earlier clinical trials. Trabectedin is confirmed as an active and safe agent for the treatment of advanced STS patients who have failed to standard therapies. Patients treated beyond 6 cycles of trabectedin had a significantly better survival, pointing out a potential role of maintenance treatment. An exhaustive retrospective study collecting the information on all patients treated with trabectedin since its approval could be very helpful to describe the outcome of patient populations in low volume centres. A prospective studies is ongoing to evaluate the efficacy of maintenance after 6 cycles.

## Competing interests

JYB, AI, BNB,ALC, IRC, NP, FD received research support and honoraria from Pharmamar. NB and PZ are employees of Pharmamar.

## Authors’ contribution

JYB: conceived the study, contributed to case collection, performed the analysis, the interpretation and wrote the primary version of the manuscript. IRC: conceived the study, contributed to case collection, contributed to the interpretation and contributed to the writing of the manuscript. ALC: contributed to case collection, contributed to the interpretation and contributed to the writing of the manuscript. FD, MR, OC, FB, FB, EB, NI, LC, PC, BNB, GD, OD, AI and NP: contributed to case collection, and contributed to the writing of the manuscript. JMC: contributed to case collection and pathology review, and contributed to the writing of the manuscript. PZ and NBa: Contributed to the concept and funding of the study, and reviewed the final version of the Ms. All authors reviewed and approved the final version of the article.

## Pre-publication history

The pre-publication history for this paper can be accessed here:

http://www.biomedcentral.com/1471-2407/13/64/prepub
